# The Cat and the Monkey

**Published:** 1915-11

**Authors:** 


**A Monthly Review of Medicine and Surgery**

EDITOR AND PUBLISHER, DR. A. L. BENEDICT, 228 Summer St., corner of Elmwood Ave., Buffalo, (Address for all communications. Please make personal and telephone calls before 1 P. M.)

Third, in age, on the western continent. Independent, but supports professional organization. News limited mainly to Western N. Y. and Penn.

Subscription $2.00 a year; $3.00 for two years in advance. Changes and errors in address or failure or duplication of delivery, should be reported immediately.

Yearly Volume 71 NOVEMBER, 1915 No. 4

### The Cat and the Monkey.

You remember the old fable of the monkey who used the cat's paws to rake hot chestnuts out of the fire. Did you ever realize that it had a practical application to medical ethics? The practice does not improve the moral status of the monkey; it is derogatory to the self respect of the cat. In quite an extensive observation, we have rarely seen the cat develop into a monkey.

